# Posterior Reversible Encephalopathy Syndrome: Clinical Features and Outcome

**DOI:** 10.3389/fneur.2020.00071

**Published:** 2020-02-14

**Authors:** Archana Hinduja

**Affiliations:** Department of Neurology, The Ohio State University Wexner Medical Center, Columbus, OH, United States

**Keywords:** posterior reversal encephalopathy syndrome, outcome, prognosis, seizures, management

## Abstract

**Background:** Posterior reversible encephalopathy syndrome (PRES) is an acute neurotoxic syndrome that is characterized by a spectrum neurological and radiological feature from various risk factors. Common neurological symptoms includes headache, impairment in level of consciousness, seizures, visual disturbances, and focal neurological deficits. Common triggering factors include blood pressure fluctuations, renal failure, eclampsia, exposure to immunosuppressive or cytotoxic agents and autoimmune disorders. The classic radiographic findings include bilateral subcortical vasogenic edema predominantly affecting the parieto-occipital regions but atypical features include involvement of other regions, cortical involvement, restricted diffusion, hemorrhage, contrast enhancement. This review is aimed to summarize the updated knowledge on the typical and atypical clinical and imaging features, prognostic markers and identify gaps in literature for future research.

**Methods:** Systematic literature review using PUBMED search from 1990 to 2019 was performed using terms PRES was performed.

**Results:** While clinical and radiographic reversibility is common, long-standing morbidity and mortality can occur in severe forms. In patients with malignant forms of PRES, aggressive care has markedly reduced mortality and improved functional outcomes. Although seizures were common, epilepsy is rare. Various factors that have been associated with poor outcome include altered sensorium, hypertensive etiology, hyperglycemia, longer time to control the causative factor, elevated C reactive protein, coagulopathy, extensive cerebral edema, and hemorrhage on imaging.

**Conclusion:** Large prospective studies that accurately predict factors that are associated with poor outcomes, determine the pathophysiology, and targeted therapy are required.

## Introduction

Posterior reversible encephalopathy syndrome (PRES) is a well-recognized entity characterized by a combination of clinical and neuroimaging findings. It was initially described by Hinchey in 1996 and subsequently has subsequently gained increasing attention (1). Key elements that are essential in its diagnosis include a combination of clinical features, radiological findings in the presence of various risk factors. Various neurological symptoms includes headache, impairment in level of consciousness, seizures, visual abnormalities, nausea, vomiting, and focal neurological deficits (1). On neuroimaging, it is characterized by vasogenic edema involving the cortical/subcortical regions which is bilateral affecting the parietal and occipital regions, followed in frequency by involvement of other regions (1–5). Its recognition has improved markedly over the last few decades with increased availability of magnetic resonance imaging (MRI) (6).

Common triggering factors of PRES include blood pressure fluctuations, preeclampsia/eclampsia, renal failure, cytotoxic agents, and autoimmune conditions (7, 8). Recently, several etiologies and atypical features have being increasingly recognized. Early recognition is crucial, as timely management of its precipitating factor is required to achieve reversibility (7). In severe cases, aggressive supportive care in the intensive care unit (ICU) is required. Despite a common myth of its benign course and reversibility in terms of both clinically and radiological aspects, permanent brain damage, severe functional impairments, and mortality have been reported (7, 9, 10). The aim of this review is to provide an updated knowledge of the clinical features and functional outcome in patients with PRES.

## Materials and Methods

Systematic literature review using PUBMED search from 1990 to 2019 was performed using terms posterior reversible encephalopathy syndrome, hypertensive encephalopathy, reversible posterior cerebral edema syndrome, clinical features, imaging, prognosis, seizures, epilepsy, pathophysiology, outcome. Articles in english, cases, case series, retrospective studies, meta-analysis, reviews, book chapters related to PRES were included. Articles were selected primarily based on the relevance to the topic and the information it provided. Articles related to both adults and pediatric PRES were included. Articles that provided duplicate information were deleted. Majority of studies were single center retrospective reports ranging from small to large sample sizes. However, there is paucity of data on its true incidence and prevalence in large patient populations.

### Epidemiology

Following its initial description in 1996, its recognition from other etiologies has increased exponentially over the last decade. These reports are in the form of case reports, case series, and large retrospective studies from large institutions. It has been reported in all age groups starting from infancy to older adults, but most frequently affects the young or middle-aged adults (7, 11). It appears to have a female predominance, even after exclusion of patients with eclampsia (6, 8, 12). While the incidence of PRES in the general population is unknown, its incidence in a selected cohort of patients is available. The incidence of PRES in pediatric population is 0.04% (13) and in pediatric intensive care unit is 0.4% (14). Among adults, it has been reported in 2.7–25% of patients following bone marrow transplantation (15–17), 0.4% following solid organ transplantation (18), 0.84% of patients with end stage renal disease is 0.84% (19) and 0.69% of patients with systemic lupus erythematosus (20).

### Pathophysiology

Several theories have been proposed in the pathogenesis of PRES (8, 21). They all lead to activation and injury of the endothelium, activation of the immune system and release of cytokines (22). The leading theory, is the “vasogenic theory,” that postulated that rapidly developing hypertension with failure of cerebral autoregulation causing breakdown of blood brain barrier and secondary vasogenic edema. When the rise in blood pressure is rapid and severe, the auto regulatory response is insufficient resulting in hyperperfusion, and extravasation of plasma and macromolecules. The relative lack of sympathetic innervation in the posterior circulation is the likely mechanism for the preferential involvement of the posterior part of the brain from PRES. The hypertension hyperperfusion theory is supported by the fact that prompt treatment of hypertension leads to rapid clinical and radiological improvement (8). In a retrospective study that compared the involvement of posterior circulation exclusively by PRES from anterior circulation involvement by PRES (either exclusive or in addition to the posterior circulation) the mean blood pressure was higher in the latter group (*p* < 0.01), which supports the vasogenic theory (23). The density of the autonomic nervous system is higher in the anterior circulation providing better control of autoregulation, but an abrupt massive rise in blood pressures can make the anterior circulation susceptible. However, this theory does not explain the mechanism in patients with borderline hypertension and normotensives. The “neuropeptide” theory has postulated that release of potent vasoconstrictors, such as endothelin-1, prostacyclin, and thromboxane A2 causes vasospasm, ischemia and cerebral edema (24). To support this, both invasive and non-invasive studies have demonstrated irregularities of the cerebral vasculature and hypoperfusion on perfusion studies (25). PRES has been observed in patients with normal blood pressure, patients in the upper limit of autoregulation or did not have blood pressure fluctuations and patients with hypotension (8, 26). To support this, endothelial dysfunction from the cytotoxic effects from infection, sepsis, chemotherapeutic agents, and immunogenic effects from autoimmune disorders, immune suppressive agents have been proposed. The “cytotoxic theory” suggests that the primary insult is from endogenous stimulants or exogenous toxins like chemotherapy or immunosuppressive agents and the “immunogenic theory” has postulated that T-cell activation and cytokine release causes endothelial dysfunction and deranged autoregulatory response (21, 27). Recently, activation of arginine vasopressin (AVP) axis by increase in AVP secretion or AVP receptor density has been postulated in the development of PRES (28). Activation of cerebral AVP receptors (V1aR) leads to cerebral vasoconstriction, endothelial dysfunction and cerebral ischemia and activation of the peripheral (renal) receptors (V2R) may potentially lead to the development of hypertension, renal impairment and is responsible for the symptoms and complications of PRES. In susceptible patients, pronounced fluctuations in blood pressure rather than the absolute increase in blood pressure and hypotension from sepsis may precede the occurrence of PRES. In certain cases, several factors might be coexistent. For example in patients with renal failure, it is unclear if renal dysfunction is an independent factor or the comorbid hypertension, autoimmune disease, or other systemic conditions are the culprit. Despite the heterogeneity in its etiologies and proposed mechanisms, PRES is a downstream effect that is characterized by a combination of clinical and radiological features. It is important to differentiate these features from other alternative conditions.

### Risk Factors

PRES was initially observed in patients with hypertension and subsequently recognized in the normotensive and septic patients. Common risk factors associated with PRES include abrupt elevations of blood pressure, impaired renal function, preeclampsia/eclampsia, autoimmune diseases, infection, transplantation, and chemotherapeutic agents. The extensive list of risk factors associated with PRES is described in Table 1.

**Table 1:** Risk factors associated with posterior reversible encephalopathy syndrome.

**Preeclampsia, Eclampsia**	
Blood pressure fluctuations	Hypertension, dysautonomia e.g., guillian barre syndrome, post carotid endarterectomy with reperfusion syndrome, induced hypertension—treatment of vasospasm in subarachnoid hemorrhage, drug withdrawal—clonidine, triamterene, prazosin, stimulant drugs—phenylpropanolamine, ephedrine, pseudoephedrine, amphetamine, cocaine
Infection	Sepsis, shock
Renal diseases	Hemolytic uremic syndrome, acute glomerulonephritis, acute and chronic renal failure, parenchymal diseases, renal artery stenosis
Immunosuppressive drugs, chemotherapeutic agents	Cyclosporin A, tacrolimus/FK-506, methotrexate, sirolimus, interferon alpha, intravenous immunoglobulin, cisplatin, vincristine, cytarabine, gemcitabine, oxaliplatin, ipilimumab, bortezomib, thalidomide, apatinib, rituximab, erythropoietin, interleukin, antiretroviral therapy in HIV- indinavir, ivabradine, granulocytic stimulating factor, tyrosine kinase inhibitors—pazopanib, sorafenib, sunitinib, high dose steroids (methylprednisolone), post solid organ, or bone marrow transplantation, tumor lysis syndrome
Autoimmune disorders	Systemic lupus erythematosus, sjogren's disease, vasculitis, scleroderma, cryoglobulinemia, polyarteritis nodosa, wegner's granulomatosis, behcet's disease, hashimoto's thyroiditis, primary sclerosing cholangitis
Hematological disorders	Thrombotic thrombocytopenic purpura, henoch-schonlein purpura, leukemia and lymphomas, sickle cell anemia, hemolytic uremic syndrome
Endocrine disorders	Pheochromocytoma, primary aldosteronism
Electrolyte disturbances	Hypercalcemia, hypomagnesemia
Others	Acute porphyria, blood transfusion, lithium

### Clinical Features

The symptoms of PRES are often non-specific and manifest acutely or subacutely over several hours or days (7). However, continued progression over several weeks is uncommon. Most of the literature related to PRES comes from retrospective observational studies and the frequency of these symptoms is dependent on the sample size evaluated and the precipitating factors. The symptoms are highly non-specific, with encephalopathy and seizures being the most common symptoms followed by visual disturbances, headache, and focal neurological deficits (8, 29).

#### Common Clinical Manifestations

Encephalopathy of varying grades has been reported in 28–94% of patients with PRES (7–9). These range from mild confusion, cognitive deficits, somnolence, stupor, and coma. It is one of the major driving factors for admission to the intensive care unit due to their risk of respiratory failure from worsening mental status (30).

Seizures commonly occur early in the disease course, and are observed in 74–87% of patients (7, 8). Various types of seizures can occur in these patients. These include generalized tonic clonic (54–64%), partial seizures (3–28%), and status epilepticus (3–17%). The most common type is the generalized tonic-clonic seizures (31–34). These typically occur within the first 24–48 h of presentation (31, 32). It is not uncommon to have serial seizures during the acute phase (32). On certain occasions, status epilepticus may be the presenting symptom of PRES (35). In the majority, seizures are terminated spontaneously or from use of antiepileptic therapy. It is common to have provoked seizures from recurrent PRES or other provoking factors around the acute phase (31). Despite a high frequency of seizures during the acute phase, the long-term risk of unprovoked seizures is infrequent and epilepsy is rare (31). PRES related epilepsy has been reported in 1–3.9% of patients (31, 36). Patients with widespread involvement from PRES on imaging are more likely to have a single seizure upon presentation but this does not translate to worse outcomes (32). Several studies have revealed lack of correlation between the imaging findings, grade of PRES, the number of lobes affected from PRES, cortical involvement and presence of hemorrhage with predilection of seizures (33, 34). There is lack of correlation between various EEG patterns and MRI findings (32, 33). Recurrent seizures have been observed in patients with atypical PRES (37). MRI in patients with long-term PRES related epilepsy might be normal, have atrophic changes or hippocampal sclerosis (33, 34, 36). Although half of the patients have persistent abnormalities on follow up imaging, recurrent seizures, and epilepsy is rare (34). The occurrence of seizures during the acute phase has not been associated with increased length of hospital stay, morbidity, mortality, or nursing home placement upon discharge (31, 32, 34). It is possible that the occurrence of seizures might have played a role in the prompt identification of this diagnosis that translated to aggressive care and improved outcomes.

Headache has been reported in 50% of patients (9). It is usually dull, diffuse, and gradual in onset. A thunderclap headache in the context of PRES should prompt us to evaluate associated for reversible cerebral vasoconstriction syndrome (RCVS) by additional imaging studies. PRES is reported in 9% of RCVS cases and conversely RCVS angiographic changes have been described in PRES (38, 39).

Varying degrees of visual symptoms have been reported in 39% of patients (7, 9). These include decreased visual acuity, diplopia, visual field deficits, cortical blindness, color vision abnormality, and visual hallucinations. Fundoscopic examination is often unremarkable but papilledema with flame shaped retinal hemorrhages and exudates have been observed in the setting of hypertension.

Focal neurological deficits like aphasia, hemiparesis have been observed in 19% of patients (9).

#### Uncommon Clinical Manifestations

In rare occasions, myelopathic symptoms and signs from spinal cord involvement have been demonstrated (40). Other uncommon presentations include abulia, agitation, delusions, ophisthotonus, optic ataxia, ocular apraxia, and simultagnosia (41–44).

### Neurodiagnostics

#### Serology

Various serological abnormalities have been observed in patients with PRES. Patients with PRES from deranged electrolytes like hypomagnesemia, hypercalcemia, and renal failure have abnormal electrolytes and renal function tests, respectively. In patients with PRES from underlying malignancy and preeclampsia, elevated lactate dehydrogenase levels (LDH) have been reported, which supports endothelial dysfunction as the possible mechanism (45). Elevated serum LDH levels have correlated with larger and more diffuse lesions on imaging (*p* < 0.01) (46). Elevated C reactive protein (CRP) levels have been associated with increased mortality in PRES patients (47). Low serum albumin levels have been observed in 70% of patients (48, 49). Serum albumin levels may contribute to the development of edema, but its correlation with the type of edema has been inconsistent across various studies (48, 49).

#### Cerebrospinal Fluid

Cerebrospinal fluid (CSF) protein levels are elevated in 70% of patients (50, 51). A direct correlation between elevated protein levels with the extent and topographical distribution of cerebral edema was observed (50, 51). However, CSF pleocytosis is rare and its presence is a marker of infarction or hemorrhage (50, 51).

#### Electroencephalogram

Data on various electroencephalographic (EEG) patterns primarily comes from several retrospective studies. In these patients, EEG was obtained based on the clinical judgment of the treating physician at variable time frames from symptom onset. Common indications of EEG in these studies were seizures and varying degrees of encephalopathy for exclusion of non-convulsive status epilepticus (52). EEG can help identify patients with ictal or epileptiform activity. Various EEG patterns have been observed in patients with and without seizures related to PRES (31, 33, 34). The most common pattern in a patient with PRES related seizure was generalized slowing followed by focal slowing, epileptiform discharges, periodic lateralized epileptiform discharges, and normal patterns. There is great variability between EEG findings and the development of epilepsy (31, 33, 36). Focal findings on EEG are commonly observed in patients with focal seizures (32). In a prospective study, non-convulsive seizures were associated with the presence of periodic discharges (*p* = 0.0002) (53). Both non-convulsive seizures and periodic discharges are usually either lateralized or bilateral independent and predominant in the posterior head regions. However, there was lack of correlation between non-convulsive seizures and periodic discharges with the clinical presentation. Restricted diffusion involving the cortex on MRI was frequent in patients with periodic discharges and non-convulsive seizures group (*p* < 0.001). A high likelihood of poor outcome in patients with non-convulsive seizures and periodic discharges has been observed (*p* < 0.04). A brief overview of various EEG patterns observed in patients with PRES is described in Table 2.

**Table 2:** EEG findings in patients with PRES.

**EEG in patients with seizures at presentations**
Generalized slowing with or without focal slowing
Generalized slowing with additional EEG abnormalities—Epileptiform discharges, Electrographic seizures, Periodic lateralized epileptiform discharges
Focal slowing with or without epileptiform discharge
Periodic lateralized epileptiform discharges
Electrographic status epilepticus
Normal
**EEG in patients without seizures at presentation**
Generalized slowing with or without additional focal slowing
Periodic lateralized epileptiform discharges
Focal sharp waves
Normal
**EEG prior to discontinuation of antiepileptic drugs**
Normal
Generalized
slowing
Focal slowing

#### Neuroimaging

Brain imaging is the cornerstone in confirming a diagnosis of PRES. Although vasogenic edema can be visualized on non-contrast computed tomography (CT) in some patients, brain MRI, especially the T2-weighted and fluid attenuated inversion recovery (FLAIR) sequences are much more sensitive (6). Currently there is no gold standard diagnostic test. When compared to T2 weighted images, FLAIR helps in detecting cortical and a subcortical lesion related to PRES and is an important sequence in establishing its diagnosis (54). Diffusion weighted imaging combined with apparent diffusion coefficient (ADC) mapping sequences are helpful in differentiating cytotoxic from vasogenic edema and thus may aid in the differentiating PRES form ischemic lesions (55, 56).

The classic imaging patterns usually reveals vasogenic edema that involves the parieto-occipital regions, bilateral, subcortical, and symmetrical in appearance. Various patterns have been described in literature (Table 3). These include: parietooccipital pattern, holohemispherical watershed pattern, and superior frontal sulcus pattern (6, 57). Occasionally, the edema may have a central-variant (brainstem) pattern that affects the brainstem, basal ganglia, posterior limb of internal capsule, cerebellum, periventricular regions, and lacks cortical and subcortical involvement (58). Cerebral edema in these patients has been classified into different grades as mild, moderate, and severe (59). Mild PRES was defined as cortical or subcortical white matter edema without hemorrhage, mass effect, herniation, and minimal involvement of one of the group—cerebellum, brain stem, or basal ganglia. Moderate PRES was defined as confluent edema extending from cortex to deep white matter without extension to the ventricular margin or mild involvement of two of the group—cerebellum, brainstem, or basal ganglia. Mild mass effect but no herniation or midline shift, presence of parenchymal hemorrhage was classified as moderate. Severe PRES was defined as confluent edema extending from the cortex to the ventricle, edema, or hemorrhage causing midline shift or herniation or involvement of three of the group—cerebellum, brainstem, or basal ganglia. Patients with worsening degree of cerebral edema have worse outcomes (60). Atypical findings include unilateral involvement, restricted diffusion, intracerebral hemorrhage, microhemorrhages, and contrast enhancement (59). Lesions may be asymmetric in about 50% of cases and unilateral in rare occasions (8, 61). Small areas of restricted diffusion compared to the large areas of vasogenic edema are seen in 30% of patients (12). The presence of restricted diffusion may be associated with incomplete recovery (62). Varying degrees of PRES related hemorrhage have been observed in 10–30% of cases (12, 63). These range from minute focal hemorrhage (<5 mm), sulcal SAH, focal hematoma and microhemorrhages on susceptibility-weighted imaging (SWI) (63). The greatest frequency of hemorrhage was seen in patients after allo-BMT and in patients with coagulopathy (12, 63). The correlation between hemorrhage and the severity of edema has been inconsistent across studies (12, 59). Susceptibility weighted imaging sequence helps in differentiating frank hemorrhage from microhemorrhage that has been observed in certain cases of PRES, however its clinical relevance in patients with PRES is unknown (64). About 40% of patients have contrast enhancement on T1-weighted imaging, the most common being leptomeningeal and leptomemnigeal plus cortical (59, 60). There was no correlation between contrast enhancement with age, imaging severity, and outcome (60). On cerebral angiography or MR angiography studies, moderate to severe vessel irregularity suggestive of vasoconstriction and vasodilation is seen in more than 80% of patients (25). On follow up there is reversal of spasm in the majority with residual spasm in a few patients (65). Magnetic venograms are normal in these patients. On MR spectroscopy, the N-acetylaspartamine (NAA)/creatine (Cr), and NAA/choline-containing compounds (Cho) were significantly lower than healthy controls at initial presentation and on 2 weeks follow up and may assist in differentiating cerebral edema from ischemia (65, 66). MR Perfusion and single-photon emission CT (SPECT), technetium Tc99m-hexamethylpropyleneamine oxime (Tc99m-HM-PAO) have demonstrated decreased cerebral blood flow from hypoperfusion in the majority (67). However, in certain cases, hyperperfusion may be observed early in the disease course (67).

**Table 3:** Imaging findings in patients with PRES.

**Common features**
Vasogenic cerebral edema Parieto-occipital pattern Holohemispherical watershed distribution Frontal and temporal lobe involvement Subcortical white matter Bilateral, frequently symmetric pattern Hyperintense T2 weighted and FLAIR sequences Isointense, hypointense, or hyperintense lesions on DWI Increased ADC values reflective of vasogenic cerebral edema
**Uncommon**
Brainstem (Central) variant Unilateral PRES Contrast enhancement Microhemorrhages Intracerebral hemorrhages Sulcal SAH Decreased ADC values indicative of ischemia
**Grades of cerebral edema**
Mild
Moderate
Severe

### Diagnosis

The spectrum of clinical features, vasogenic cerebral edema and various risk factors are crucial in making a diagnosis of PRES (8). It has a fairly rapid onset and may have a stuttering course. More than 90% of patients have typical radiological and clinical features (27). In a retrospective study, seizures, encephalopathy, visual disturbances, hypertension, renal failure, chemotherapy were the best clinical predictors of PRES, while headache, immunosuppression, and autoimmune disorders were not useful in making a clinical diagnosis of PRES (68). Brain imaging in the context of clinical features to exclude other diagnostic condition is crucial in making an accurate diagnosis. About 95% of patients have cortical-subcortical appearance of vasogenic edema, irrespective of small foci of cytotoxic edema on diffusion weighted imaging, contrast enhancing foci or microhemorrhages (6, 8, 59). More than 95% have involvement of the parieto-occipital region and high precentral/posterior frontal region that is disproportionate to the rest of the brain (59, 67). Recently the PRES early warning scoring (PEWS) scale which consisted of (1) risk factors, (2) clinical features and (3) EEG features has improved the prediction of PRES early in suspected cases, with a high index of suspicion in patients with a score of 10 points or higher (65, 69).

#### Differential Diagnosis

Differentiating atypical features of PRES like central PRES, hemorrhagic PRES from other causes like toxic leucoencephalopathy, meningoencephalitis, central/extra pontine myelinolysis, lupus cerebritis, malignancy, hypoxic ischemic encephalopathy requires a thorough review of risk factors, additional testing and follow up imaging (58, 70). In acute ischemic stroke, decreased ADC points toward cytotoxic edema from stroke than PRES. In central/extra pontine myelinolysis the ADC is raised and the rapid correction of electrolyte abnormalities should assist in making its diagnosis. Besides, there is enhancement in the subacute phase on follow up post contrast MRI. Differentiating tumor from PRES is based on time frame of symptom involvement and lack of resolution on follow up imaging. Gliomatosis cerebri is isointense or hypointense on T1-weighted image and hyperintense on T2-weighted image and on MR spectroscopy there is elevated choline/NAA peak. Hypoxic ischemic encephalopathy can be differentiated by the history, gyriform pattern of restricted diffusion predominantly involving the cortex and lack of resolution on follow up imaging. Infectious encephalitis especially rhombencephalitis may be made by clinical history and clinical examination. Reversible cerebral vasoconstriction syndrome may be differentiated by classic thunderclap headache in the presence of known triggers and vasoconstriction of the vessels. In certain cases, this may coexist with PRES. Acute hepatic encephalopathy is differentiated by the history of chronic liver disease, hyperintensity on FLAIR with possibly restricted diffusion in both thalami, posterior limb of internal capsule and periventricular white matter.

### Management

Management is primarily supportive and guided by expert consensus. Prompt recognition is the key as timely removal of the precipitating factor is important to achieve favorable outcomes (1, 7). Currently there are no randomized trials on various interventions have been conducted in these patients. About 70% of patients require ICU care for aggressive management of their symptoms (30). Common indications for transfer to the ICU include encephalopathy, seizures, and status epilepticus (30). The following steps should be performed (1, 52):

1. Removal or reduction of the triggering factor (withdrawal of cancer chemotherapy or immunosuppressive agents). In patients with PRES related to cancer chemotherapy or immunosuppressive agents, long term management of immunosuppressive agents and chemotherapy remains a challenging issue and should be individualized.
2. Supportive care with hydration, correction of electrolyte disturbances.
3. Monitoring of airway and ventilation. Intubation may be required in patients with altered mental status.
4. In pregnant women, prompt delivery should be considered.
5. In patients with renal failure, prompt dialysis should be performed.
6. In patients with acute hypertension, gradual reduction of blood pressure should be performed (no more than 20–25% in the first few hours) to avoid the risk of cerebral, coronary, and renal ischemia (71). The goal is to maintain mean arterial pressure between 105 and 125 mm Hg. Intravenous agents are preferred to avoid fluctuations of blood pressure and the choice of agents is left to the discretion of the physician. Continuous infusions are frequently required to avoid fluctuations of blood pressure and achieve the goal blood pressure. First line agents include nicardipine, labetalol, nimodipine, and second line agents include sodium nitroprusside, hydralazine, and diazoxide. Avoid angiotensin converting enzyme inhibitors in pregnant women. Fenoldopam mesylate, a selective dopamine 1 agonist that produces renal vasodilation may improve the renal oxygen supply/demand ratio and prevent renal failure. In patients subarachnoid hemorrhage with PRES from induced hypertension for vasospasm gradual reduction of blood pressure is crucial for neurological improvement (61).

Treatment of status epilepticus

1. Intravenous anticonvulsants (first line with diazepam, second line with forphenytoin, phenobarbital.
2. In refractory cases propofol, pentobarbital, midazolam may be used.
3. Continuous EEG monitoring may be considered.
4. In pregnant women, magnesium sulfate is indicated to prevent seizures. It has cerebral vasodilatory effects and reduces blood vessel permeability.

Although seizures are common long-term data on risk of recurrent seizures and epilepsy is limited due to lack of large population based studies. Currently, there are no standard guidelines for management of PRES related seizures and treatment with antiepileptic agents must be made based on individual basis. Antiepileptic drugs are frequently prescribed to patients with seizures. As epilepsy is rare long-term antiepileptic medications are not warranted in majority of these patients. There is often a dilemma on the optimal duration of antiepileptics. The most common antiepileptics that have been used during hospitalization include benzodiazepines, levetiracetam and phenytoin and upon discharge levetiracetam and phenytoin, with majority of them on a single agent. Since seizures are uncommon out of the acute phase, antiepileptic agents may be quickly tapered. In a single center study, the median duration of antiepileptic agents was 3 months (IQR 2–7 months). The overall prognosis of both generalized and focal seizures in PRES is benign. Besides, not all patients with seizures have been treated with antiepileptic agents and none of these patients developed recurrent seizures (31, 32). It is unclear if antiepileptic agents play a role on the risk of subsequent seizures and epilepsy in these patients. If antiepileptic agents are started, discontinuation following resolution of PRES should be considered, once there is adequate control of risk factors, and absence of factors that might substantially lower the seizure threshold.

### Complications

#### Recurrent PRES

Recurrent PRES has been observed in 4% of patients in retrospective studies (72). It is not uncommon for patients to have recurrent episodes of PRES from recurrence of risk factors like sickle cell crisis, autoimmune conditions, hypertensive crisis, renal failure, and multiorgan failure.

#### Malignant PRES

The term malignant PRES has been defined based on clinical criteria (Glasgow Coma Score <8 and clinical decline despite standard medical management for elevated intracranial pressure) and radiological criteria (edema with mass effect, intracerebral hemorrhage exerting mass effect, effacement of basal cisterns, transtentorial, tonsillar, or uncal herniation) (73).

Management of malignant PRES requires aggressive supportive care. In a case series, besides routine care like mechanical ventilation, transfusion of blood products for reversal of coagulopathy, steroids for autoimmune disorders, intracranial pressure monitoring is required in patients with GCS of ≤8 (73). Various interventions that have been undertaken in patients with raised ICP include osmotherapy, draining of cerebrospinal fluid by external ventricular drain, craniectomy and evacuation of hematoma. Due to aggressive care, no fatalities were observed in patients with severe or hemorrhagic PRES variants compared to historic reports of 16–29% (63, 74). All patients achieved favorable functional outcomes based on the mRS (modified Rankin Score of 1–2) on long term follow up (73).

In patients with acute obstructive hydrocephalus, an external ventricular drain placement may be required for management (75).

### Prognosis

Although PRES was initially described as a benign entity that was reversible with a good outcome, mortality has been observed in 19% of patients and functional impairments of varying degree have been reported in 44% of patients (9, 10). Certain deficits that require long-term care include epilepsy and motor deficits.

PRES is an acute neurotoxic syndrome and the prognosis is highly dependent on the etiological factor. Studies have reported that patients with preeclampsia-eclampsia have less severe cerebral edema, hemorrhage, contrast enhancement with a tendency for complete resolution on imaging and good functional outcome (10, 29). A recent systemic review and meta-analysis which included 448 PRES patients showed good outcomes in patients with PRES related to pre-eclampsia/eclampsia (*p* < 0.00001) (76). Other factors that have been associated with poor outcome include severe encephalopathy, hypertensive etiology, hyperglycemia, neoplastic etiology, longer time to control the causative factor, the presence of multiple comorbidities, elevated CRP, low CSF glucose, and coagulopathy (9, 10, 47, 77). Residual structural lesions have been observed in 40% of cases on follow up imaging (12). Various imaging features that are associated with poor outcome include corpus callosum involvement, extensive cerebral edema or worsening imaging severity, hemorrhage, subarachnoid hemorrhage, and restrictive diffusion on imaging (47, 60, 76–78). The type, location and severity of hemorrhage that is associated with poor outcome are inconsistent across various studies (47, 76, 79). While small hemorrhages do not have an impact on outcome, multiple or massive hemorrhages might be associated with poor outcome. Several studies have demonstrated correlation between the degree of hypertension with clinical outcome and severity of edema on imaging. Interestingly, while the severity of edema on MRI correlated with clinical outcomes, the presence or patterns of gadolinium based contrast enhancement did not correlate with functional outcomes (60). To summarize, although there are several associations, identifying a single predictor of outcome has been challenging in these patients.

## Future Directions

Recent data from animal studies have demonstrated blood brain barrier disruption as a possible mechanism for development of vasogenic cerebral edema from acute hypertension and thus may be a target of future intervention (80). Besides, based on the recent AVP theory, suppression of AVP the use of vaptans might play a role in the treatment of PRES. Currently, there is paucity of data on its clinical implications in PRES.

## Conclusion

Currently, the available data on outcomes are from single institutions with paucity of data from long-term epidemiological studies. Its heterogeneous nature limits its ability to generalize. PRES has a favorable prognosis in general, but fatalities can occur. A standardized algorithm that incorporates the clinical, etiological, serological markers, imaging features with various comorbidities and will assist in future studies. Various pathophysiological mechanisms need to be explored at bench side to determine reliable laboratory and imaging markers and therapeutic interventions in order to improve functional outcomes are warranted.

## Author Contributions

The author confirms being the sole contributor of this work and has approved it for publication.

### Conflict of Interest

The author declares that the research was conducted in the absence of any commercial or financial relationships that could be construed as a potential conflict of interest.

## References

[1] Hinchey J Chaves C Appignani B Breen J Pao L Wang A. A reversible posterior leukoencephalopathy syndrome. N Engl J Med. (1996) 334:494–500. 10.1056/NEJM199602223340803 8559202

[2] Schwartz RB Jones KM Kalina P Bajakian RL Mantello MT Garada B. Hypertensive encephalopathy: findings on CT, MR imaging, and SPECT imaging in 14 cases. AJR Am J Roentgenol. (1992) 159:379–83. 10.2214/ajr.159.2.1632361 1632361

[3] Schwartz RB Bravo SM Klufas RA Hsu L Barnes PD Robson CD. Cyclosporine neurotoxicity and its relationship to hypertensive encephalopathy: CT and MR findings in 16 cases. AJR Am J Roentgenol. (1995) 165:627–31. 10.2214/ajr.165.3.7645483 7645483

[4] Bartynski WS Grabb BC Zeigler Z Lin L Andrews DF. Watershed imaging features and clinical vascular injury in cyclosporin A neurotoxicity. J Comput Assist Tomogr. (1997) 21:872–80. 10.1097/00004728-199711000-00005 9386275

[5] Bartynski WS Zeigler Z Spearman MP Lin L Shadduck RK Lister J. Etiology of cortical and white matter lesions in cyclosporin-A and FK-506 neurotoxicity. AJNR Am J Neuroradiol. (2001) 22:1901–14. 11733324 PMC7973826

[6] Bartynski WS Boardman JF. Distinct imaging patterns and lesion distribution in posterior reversible encephalopathy syndrome. AJNR Am J Neuroradiol. (2007) 28:1320–7. 10.3174/ajnr.A0549 17698535 PMC7977645

[7] Lee VH Wijdicks EF Manno EM Rabinstein AA. Clinical spectrum of reversible posterior leukoencephalopathy syndrome. Arch Neurol. (2008) 65:205–10. 10.1001/archneurol.2007.46 18268188

[8] Fugate JE Claassen DO Cloft HJ Kallmes DF Kozak OS Rabinstein AA. Posterior reversible encephalopathy syndrome: associated clinical and radiologic findings. Mayo Clin Proc. (2010) 85:427–32. 10.4065/mcp.2009.0590 20435835 PMC2861971

[9] Legriel S Schraub O Azoulay E Hantson P Magalhaes E Coquet I. Determinants of recovery from severe posterior reversible encephalopathy syndrome. PLoS ONE. (2012) 7:e44534. 10.1371/journal.pone.0044534 23024751 PMC3443081

[10] Alhilali LM Reynolds AR Fakhran S. A multi-disciplinary model of risk factors for fatal outcome in posterior reversible encephalopathy syndrome. J Neurol Sci. (2014) 347:59–65. 10.1016/j.jns.2014.09.019 25271189

[11] Kummer S Schaper J Mayatepek E Tibussek D. Posterior reversible encephalopathy syndrome in early infancy. Klin Padiatr. (2010) 222:269–70. 10.1055/s-0030-1249031 20514606

[12] Liman TG Bohner G Heuschmann PU Endres M Siebert E. The clinical and radiological spectrum of posterior reversible encephalopathy syndrome: the retrospective Berlin PRES study. J Neurol. (2012) 259:155–64. 10.1007/s00415-011-6152-4 21717193

[13] Thavamani A Umapathi KK Puliyel M Super D Allareddy V Ghori A Epidemiology, comorbidities, and outcomes of posterior reversible encephalopathy syndrome in children in the United States. Pediatr Neurol. (2019) 103:21–6. 10.1016/j.pediatrneurol.2019.07.007 31481327

[14] Raj S Overby P Erdfarb A Ushay HM. Posterior reversible encephalopathy syndrome: incidence and associated factors in a pediatric critical care population. Pediatr Neurol. (2013) 49:335–9. 10.1016/j.pediatrneurol.2013.06.007 23916861

[15] Reece DE Frei-Lahr DA Shepherd JD Dorovini-Zis K Gascoyne RD Graeb DA. Neurologic complications in allogeneic bone marrow transplant patients receiving cyclosporin. Bone Marrow Transplant. (1991) 8:393–401. 1768975

[16] Bartynski WS Zeigler ZR Shadduck RK Lister J Pretransplantation conditioning influence on the occurrence of cyclosporine or FK-506 neurotoxicity in allogeneic bone marrow transplantation. AJNR Am J Neuroradiol. (2004) 25:261–9. 14970028 PMC7974616

[17] Bartynski WS Zeigler ZR Shadduck RK Lister J. Variable incidence of cyclosporine and FK-506 neurotoxicity in hematopoeitic malignancies and marrow conditions after allogeneic bone marrow transplantation. Neurocrit Care. (2005) 3:33–45. 10.1385/NCC:3:1:033 16159093

[18] Bartynski WS Tan HP Boardman JF Shapiro R Marsh JW. Posterior reversible encephalopathy syndrome after solid organ transplantation. AJNR Am J Neuroradiol. (2008) 29:924–30. 10.3174/ajnr.A0960 18272559 PMC8128592

[19] Canney M Kelly D Clarkson M Posterior reversible encephalopathy syndrome in end-stage kidney disease: not strictly posterior or reversible. Am J Nephrol. (2015) 41:177–82. 10.1159/000381316 25871433

[20] Lai CC Chen WS Chang YS Wang SH Huang CJ Guo WY. Clinical features and outcomes of posterior reversible encephalopathy syndrome in patients with systemic lupus erythematosus. Arthritis Care Res. (2013) 65:1766–74. 10.1002/acr.22047 23687067

[21] Granata G Greco A Iannella G Granata M Manno A Savastano E. Posterior reversible encephalopathy syndrome–Insight into pathogenesis, clinical variants and treatment approaches. Autoimmun Rev. (2015) 14:830–6. 10.1016/j.autrev.2015.05.006 25999210

[22] Chen Z Shen GQ Lerner A Gao B. Immune system activation in the pathogenesis of posterior reversible encephalopathy syndrome. Brain Res Bull. (2017) 131:93–9. 10.1016/j.brainresbull.2017.03.012 28373149

[23] Marrone LCP Martins WA Borges MT Rossi BC Brunelli JPF Vedana VM Posterior reversible encephalopathy syndrome: clinical differences in patients with exclusive involvement of posterior circulation compared to anterior or global involvement. J Stroke Cerebrovasc Dis. (2016) 25:1776–80. 10.1016/j.jstrokecerebrovasdis.2016.03.042 27103268

[24] Bartynski WS. Posterior reversible encephalopathy syndrome, part 2: controversies surrounding pathophysiology of vasogenic edema. AJNR Am J Neuroradiol. (2008) 29:1043–9. 10.3174/ajnr.A0929 18403560 PMC8118813

[25] Bartynski WS Boardman JF. Catheter angiography, MR angiography, and MR perfusion in posterior reversible encephalopathy syndrome. AJNR Am J Neuroradiol. (2008) 29:447–55. 10.3174/ajnr.A0839 18079186 PMC8118858

[26] Rabinstein AA Mandrekar J Merrell R Kozak OS Durosaro O Fugate JE. Blood pressure fluctuations in posterior reversible encephalopathy syndrome. J Stroke Cerebrovasc Dis. (2012) 21:254–8. 10.1016/j.jstrokecerebrovasdis.2011.03.011 21536456

[27] Gao B Lyu C Lerner A McKinney AM. Controversy of posterior reversible encephalopathy syndrome: what have we learnt in the last 20 years? J Neurol Neurosurg Psychiatry. (2018) 89:14–20. 10.1136/jnnp-2017-316225 28794149

[28] Largeau B Le Tilly O Sautenet B Salmon Gandonniere C Barin-Le Guellec C Ehrmann S. Arginine vasopressin and posterior reversible encephalopathy syndrome pathophysiology: the missing link? Mol Neurobiol. (2019) 56:6792–806. 10.1007/s12035-019-1553-y 30924075

[29] Liman TG Bohner G Heuschmann PU Scheel M Endres M Siebert E. Clinical and radiological differences in posterior reversible encephalopathy syndrome between patients with preeclampsia-eclampsia and other predisposing diseases. Eur J Neurol. (2012) 19:935–43. 10.1111/j.1468-1331.2011.03629.x 22248235

[30] Hinduja A Habetz K Raina SK Fitzgerald RT. Predictors of intensive care unit utilization in patients with posterior reversible encephalopathy syndrome. Acta Neurol Belg. (2017) 117:201–6. 10.1007/s13760-016-0703-5 27680733

[31] Datar S Singh T Rabinstein AA Fugate JE Hocker S. Long-term risk of seizures and epilepsy in patients with posterior reversible encephalopathy syndrome. Epilepsia. (2015) 56:564–8. 10.1111/epi.12933 25690439

[32] Kastrup O Gerwig M Frings M Diener HC. Posterior reversible encephalopathy syndrome (PRES): electroencephalographic findings and seizure patterns. J Neurol. (2012) 259:1383–9. 10.1007/s00415-011-6362-9 22189837

[33] Sha Z Moran BP McKinney AM IV Henry TR. Seizure outcomes of posterior reversible encephalopathy syndrome and correlations with electroencephalographic changes. Epilepsy Behav. (2015) 48:70–4. 10.1016/j.yebeh.2015.05.027 26071927

[34] Hinduja A Habetz K Raina SK Fitzgerald RT Sahaya K. Predictors of seizures in patients with posterior reversible encephalopathy syndrome. Epilepsy Behav. (2016) 61:97–101. 10.1016/j.yebeh.2016.05.001 27337161

[35] Kozak OS Wijdicks EF Manno EM Miley JT Rabinstein AA. Status epilepticus as initial manifestation of posterior reversible encephalopathy syndrome. Neurology. (2007) 69:894–7. 10.1212/01.wnl.0000269780.45472.16 17724292

[36] Heo K Cho KH Lee MK Chung SJ Cho YJ Lee BI. Development of epilepsy after posterior reversible encephalopathy syndrome. Seizure. (2016) 34:90–4. 10.1016/j.seizure.2015.12.005 26760446

[37] Kamiya-Matsuoka C Tummala S. Electrographic patterns in patients with posterior reversible encephalopathy syndrome and seizures. J Neurol Sci. (2017) 375:294–8. 10.1016/j.jns.2017.02.017 28320152

[38] Ducros A Boukobza M Porcher R Sarov M Valade D Bousser MG. The clinical and radiological spectrum of reversible cerebral vasoconstriction syndrome. A prospective series of 67 patients. Brain. (2007) 130:3091–101. 10.1093/brain/awm256 18025032

[39] Ducros A Fiedler U Porcher R Boukobza M Stapf C Bousser MG. Hemorrhagic manifestations of reversible cerebral vasoconstriction syndrome: frequency, features, and risk factors. Stroke. (2010) 41:2505–11. 10.1161/STROKEAHA.109.572313 20884871

[40] de Havenon A Joos Z Longenecker L Shah L Ansari S Digre K. Posterior reversible encephalopathy syndrome with spinal cord involvement. Neurology. (2014) 83:2002–6. 10.1212/WNL.0000000000001026 25355822

[41] Nishie M Kurahashi K Ogawa M Yoshida Y Midorikawa H. Posterior encephalopathy subsequent to cyclosporin A presenting as irreversible abulia. Intern Med. (2003) 42:750–5. 10.2169/internalmedicine.42.750 12924507

[42] Kumar N Singh R Sharma N Jain A. Atypical presentation of posterior reversible encephalopathy syndrome: two cases. J Anaesthesiol Clin Pharmacol. (2018) 34:120–2. 10.4103/0970-9185.173351 29643636 PMC5885427

[43] Brown CH Feng AJ Cruz E. Ocular dysfunctions presenting in tacrolimus-induced posterior reversible encephalopathy syndrome: a case presentation. PMR. (2018) 10:105–11. 10.1016/j.pmrj.2017.07.079 28911995

[44] Tsai SJ Yeh CB Wang CW Mao WC Yeh TC Tai YM. Delusional infestation in a patient with posterior reversible encephalopathy syndrome. Aust N Z J Psychiatry. (2016) 50:1212–3. 10.1177/0004867416656259 27357712

[45] Fitzgerald RT Wright SM Samant RS Kumar M Ramakrishnaiah RH Van Hemert R. Elevation of serum lactate dehydrogenase at posterior reversible encephalopathy syndrome onset in chemotherapy-treated cancer patients. J Clin Neurosci. (2014) 21:1575–8. 10.1016/j.jocn.2014.03.004 24780237

[46] Gao B Liu FL Zhao B. Association of degree and type of edema in posterior reversible encephalopathy syndrome with serum lactate dehydrogenase level: initial experience. Eur J Radiol. (2012) 81:2844–7. 10.1016/j.ejrad.2011.12.010 22209526

[47] Siebert E Bohner G Liebig T Endres M Liman TG. Factors associated with fatal outcome in posterior reversible encephalopathy syndrome: a retrospective analysis of the Berlin PRES study. J Neurol. (2017) 264:237–42. 10.1007/s00415-016-8328-4 27815684

[48] Pirker A Kramer L Voller B Loader B Auff E Prayer D. Type of edema in posterior reversible encephalopathy syndrome depends on serum albumin levels: an MR imaging study in 28 patients. AJNR Am J Neuroradiol. (2011) 32:527–31. 10.3174/ajnr.A2332 21252042 PMC8013074

[49] Gao B Yu BX Li RS Zhang G Xie HZ Liu FL. Cytotoxic edema in posterior reversible encephalopathy syndrome: correlation of MRI features with serum albumin levels. AJNR Am J Neuroradiol. (2015) 36:1884–9. 10.3174/ajnr.A4379 26138140 PMC7965038

[50] Ellis CA McClelland AC Mohan S Kuo E Kasner SE Zhang C. Cerebrospinal fluid in posterior reversible encephalopathy syndrome: implications of elevated protein and pleocytosis. Neurohospitalist. (2019) 9:58–64. 10.1177/1941874418802061 30915182 PMC6429675

[51] Datar S Singh TD Fugate JE Mandrekar J Rabinstein AA Hocker S. Albuminocytologic dissociation in posterior reversible encephalopathy syndrome. Mayo Clin Proc. (2015) 90:1366–71. 10.1016/j.mayocp.2015.07.018 26349950

[52] Servillo G Bifulco F De Robertis E Piazza O Striano P Tortora F. Posterior reversible encephalopathy syndrome in intensive care medicine. Intensive Care Med. (2007) 33:230–6. 10.1007/s00134-006-0459-0 17119920

[53] Bastide L Legros B Rampal N Gilmore EJ Hirsch LJ Gaspard N. Clinical correlates of periodic discharges and nonconvulsive seizures in posterior reversible encephalopathy syndrome (PRES). Neurocrit Care. (2018) 29:481–90. 10.1007/s12028-018-0548-2 29949000

[54] Casey SO Sampaio RC Michel E Truwit CL. Posterior reversible encephalopathy syndrome: utility of fluid-attenuated inversion recovery MR imaging in the detection of cortical and subcortical lesions. AJNR Am J Neuroradiol. (2000) 21:1199–206. 10954269 PMC8174901

[55] Provenzale JM Petrella JR Cruz LC Jr Wong JC Engelter S Barboriak DP. Quantitative assessment of diffusion abnormalities in posterior reversible encephalopathy syndrome. AJNR Am J Neuroradiol. (2001) 22:1455–61. 11559490 PMC7974581

[56] Covarrubias DJ Luetmer PH Campeau NG. Posterior reversible encephalopathy syndrome: prognostic utility of quantitative diffusion-weighted MR images. AJNR Am J Neuroradiol. (2002) 23:1038–48. 12063238 PMC7976914

[57] Fischer M Schmutzhard E. Posterior reversible encephalopathy syndrome. J Neurol. (2017) 264:1608–16. 10.1007/s00415-016-8377-8 28054130 PMC5533845

[58] McKinney AM Jagadeesan BD Truwit CL Central-variant posterior reversible encephalopathy syndrome: brainstem or basal ganglia involvement lacking cortical or subcortical cerebral edema. AJR Am J Roentgenol. (2013) 201:631–8. 10.2214/AJR.12.9677 23971457

[59] McKinney AM Short J Truwit CL McKinney ZJ Kozak OS SantaCruz KS. Posterior reversible encephalopathy syndrome: incidence of atypical regions of involvement and imaging findings. AJR Am J Roentgenol. (2007) 189:904–12. 10.2214/AJR.07.2024 17885064

[60] Karia SJ Rykken JB McKinney ZJ Zhang L McKinney AM. Utility and significance of gadolinium-based contrast enhancement in posterior reversible encephalopathy syndrome. AJNR Am J Neuroradiol. (2016) 37:415–22. 10.3174/ajnr.A4563 26564441 PMC5584787

[61] Dhar R Dacey R Human T Zipfel G. Unilateral posterior reversible encephalopathy syndrome with hypertensive therapy of contralateral vasospasm: case report. Neurosurgery. (2011) 69:E1176–81; E81. 10.1227/NEU.0b013e318223b995 21971491

[62] Moon SN Jeon SJ Choi SS Song CJ Chung GH Yu IK. Can clinical and MRI findings predict the prognosis of variant and classical type of posterior reversible encephalopathy syndrome (PRES)? Acta Radiol. (2013) 54:1182–90. 10.1177/0284185113491252 23858507

[63] Hefzy HM Bartynski WS Boardman JF Lacomis D. Hemorrhage in posterior reversible encephalopathy syndrome: imaging and clinical features. AJNR Am J Neuroradiol. (2009) 30:1371–9. 10.3174/ajnr.A1588 19386731 PMC7051550

[64] McKinney AM Sarikaya B Gustafson C Truwit CL. Detection of microhemorrhage in posterior reversible encephalopathy syndrome using susceptibility-weighted imaging. AJNR Am J Neuroradiol. (2012) 33:896–903. 10.3174/ajnr.A2886 22241378 PMC7968802

[65] Sengar AR Gupta RK Dhanuka AK Roy R Das K. MR imaging, MR angiography, and MR spectroscopy of the brain in eclampsia. AJNR Am J Neuroradiol. (1997) 18:1485–90. 9296189 PMC8338157

[66] Eichler FS Wang P Wityk RJ Beauchamp NJ Jr Barker PB. Diffuse metabolic abnormalities in reversible posterior leukoencephalopathy syndrome. AJNR Am J Neuroradiol. (2002) 23:833–7. 12006287 PMC7974732

[67] Bartynski WS. Posterior reversible encephalopathy syndrome, part 1: fundamental imaging and clinical features. AJNR Am J Neuroradiol. (2008) 29:1036–42. 10.3174/ajnr.A0928 18356474 PMC8118828

[68] Faille LD Fieuws S Van Paesschen W. Clinical predictors and differential diagnosis of posterior reversible encephalopathy syndrome. Acta Neurol Belg. (2017) 117:469–75. 10.1007/s13760-017-0750-6 28144796 PMC5440491

[69] Zou LP Liu LY Li H Wang YY Liu Y Chen J. Establishment and utility assessment of posterior reversible encephalopathy syndrome early warning scoring (PEWS) scale establishment and utility assessment of PEWS scale. BMC Neurol. (2019) 19:30. 10.1186/s12883-019-1247-0 30791893 PMC6385440

[70] Rykken JB McKinney AM. Posterior reversible encephalopathy syndrome. Semin Ultrasound CT MR. (2014) 35:118–35. 10.1053/j.sult.2013.09.007 24745888

[71] Williams B Mancia G Spiering W Agabiti Rosei E Azizi M Burnier M. 2018 ESC/ESH Guidelines for the management of arterial hypertension: The Task Force for the management of arterial hypertension of the European Society of Cardiology and the European Society of Hypertension: The Task Force for the management of arterial hypertension of the European Society of Cardiology and the European Society of Hypertension. J Hypertens. (2018) 36:1953–2041. 10.1097/HJH.0000000000001940 30234752

[72] Sweany JM Bartynski WS Boardman JF “Recurrent” posterior reversible encephalopathy syndrome: report of 3 cases–PRES can strike twice! J Comput Assist Tomogr. (2007) 31:148–56. 10.1097/01.rct.0000233127.21303.b9 17259848

[73] Akins PT Axelrod Y Silverthorn JW Guppy K Banerjee A Hawk MW. Management and outcomes of malignant posterior reversible encephalopathy syndrome. Clin Neurol Neurosurg. (2014) 125:52–7. 10.1016/j.clineuro.2014.06.034 25086431

[74] Aranas RM Prabhakaran S Lee VH. Posterior reversible encephalopathy syndrome associated with hemorrhage. Neurocrit Care. (2009) 10:306–12. 10.1007/s12028-009-9200-5 19225908

[75] Catherine C Yanta C Saand AR Pilato M Chou SH. Pearls & Oy-sters: the dangers of PRES: an atypical case with life-threatening presentation. Neurology. (2019) 92:e282–5. 10.1212/WNL.0000000000006775 30643036 PMC6340383

[76] Chen Z Zhang G Lerner A Wang AH Gao B Liu J. Risk factors for poor outcome in posterior reversible encephalopathy syndrome: systematic review and meta-analysis. Quant Imaging Med Surg. (2018) 8:421–32. 10.21037/qims.2018.05.07 29928607 PMC5989088

[77] Liman TG Bohner G Endres M Siebert E. Discharge status and in-hospital mortality in posterior reversible encephalopathy syndrome. Acta Neurol Scand. (2014) 130:34–9. 10.1111/ane.12213 24329761

[78] Schweitzer AD Parikh NS Askin G Nemade A Lyo J Karimi S. Imaging characteristics associated with clinical outcomes in posterior reversible encephalopathy syndrome. Neuroradiology. (2017) 59:379–86. 10.1007/s00234-017-1815-1 28289809 PMC5565839

[79] Hinduja A Habetz K Raina S Ramakrishnaiah R Fitzgerald RT. Predictors of poor outcome in patients with posterior reversible encephalopathy syndrome. Int J Neurosci. (2017) 127:135–44. 10.3109/00207454.2016.1152966 26892843

[80] Wang Q Huang B Shen G Zeng Y Chen Z Lu C. Blood-brain barrier disruption as a potential target for therapy in posterior reversible encephalopathy syndrome: evidence from multimodal MRI in rats. Front Neurol. (2019) 10:1211. 10.3389/fneur.2019.01211 31849806 PMC6901929

